# Synthesis and crystal structure of poly[[di-μ_3_-tetra­thio­anti­monato-tris­[(cyclam)cobalt(II)]] aceto­nitrile disolvate dihydrate] (cyclam = 1,4,8,11-tetra­aza­cyclo­tetra­deca­ne)

**DOI:** 10.1107/S2056989022001074

**Published:** 2022-02-03

**Authors:** Christian Näther, Felix Danker, Wolfgang Bensch

**Affiliations:** a Institut für Anorganische Chemie, Universität Kiel, Max-Eyth.-Str. 2, D-24118 Kiel, Germany

**Keywords:** crystal structure, cobalt thio­anti­monate, layered structure, hydrogen bonding

## Abstract

Reaction of Co(ClO_4_)_2_·6H_2_O with cyclam (cyclam = 1,4,8,11-tetra­aza­cyclo­tetra­deca­ne) and Na_3_SbS_4_·9H_2_O (Schlippesches salt) in a mixture of aceto­nitrile and water leads to the formation of crystals of the title compound with the composition {[Co_3_(SbS_4_)_2_(C_10_H_24_N_4_)_3_]·2CH_3_CN·2H_2_O}_*n*_ or {[(Co-cyclam)_3_(SbS_4_)_2_]·2(aceto­nitrile)·2H_2_O}_*n*_. The crystal structure of the title compound consists of three crystallographically independent [Co-cyclam]^2+^ cations, which are located on centers of inversion, one [SbS_4_]^3−^ anion, one water and one aceto­nitrile mol­ecule that occupy general positions. The aceto­nitrile mol­ecule is disordered over two orientations and was refined using a split model. The Co^II^ cations are coordinated by four N atoms of the cyclam ligand and two *trans*-S atoms of the tetra­thio­anti­monate anion within slightly distorted octa­hedra. The unique [SbS_4_]^3−^ anion is coordinated to all three crystallographically independent Co^II^ cations and this unit, with its symmetry-related counterparts, forms rings composed of six Co-cyclam cations and six tetra­thio­anti­monate anions that are further condensed into layers. These layers are perfectly stacked onto each other so that channels are formed in which acetontrile solvate mol­ecules that are hydrogen bonded to the anions are embedded. The water solvate mol­ecules are located between the layers and are connected to the cyclam ligands and the [SbS_4_]^3−^ anions *via* inter­molecular N—H⋯O and O—H⋯S hydrogen bonding.

## Abstract 2

In the crystal structure of the title compound, the [SbS_4_]^3−^ anions are linked by the Co(cyclam) complex cations into rings, which are further connected into layers that are linked by inter­molecular hydrogen bonding *via* the water solvate mol­ecules and are arranged in such a way that cavities are formed, in which the disordered aceto­nitrile solvate mol­ecules are located.

## Chemical context

Inorganic–organic chalcogenidometallates are an important class of compounds and many such compounds have been reported in the literature (Sheldrick & Wachhold, 1988 ▸; Bensch *et al.*, 1997 ▸; Dehnen & Melullis, 2007 ▸; Wang *et al.*, 2016 ▸; Zhou, 2016 ▸; Zhu & Dai, 2017 ▸; Nie *et al.*, 2017 ▸). A large part of this family of compounds consists of thio­anti­monates, which exhibit a variety of coordination numbers that can lead to networks of different dimensionality (Jia *et al.*, 2004 ▸; Powell *et al.*, 2005 ▸; Spetzler *et al.*, 2004 ▸; Zhang *et al.*, 2007 ▸; Liu & Zhou, 2011 ▸; Engelke *et al.*, 2004 ▸; Puls *et al.*, 2006 ▸). Moreover, some of them have potential for applications, for example in the field of superionic conductors (Zhou *et al.*, 2019 ▸) or as photoconductive materials (Pienack *et al.*, 2008*a* ▸). For these reasons, we have explored such compounds over many years (Schaefer *et al.*, 2003 ▸; Stähler *et al.*, 2001 ▸; Schur *et al.*, 1998 ▸, 2001 ▸; Kiebach *et al.*, 2004 ▸; Spetzler *et al.*, 2004 ▸; Lühmann *et al.*, 2008 ▸; Pienack *et al.*, 2008*b* ▸). In the beginning, we synthesized new thio­anti­monates using elemental anti­mony, sulfur and amine mol­ecules under solvothermal conditions but later we found that many of these compounds are also available under solvothermal conditions if Schlippesches salt (Na_3_SbS_4_·9H_2_O) or NaSbS_3_ are used as reactants (Anderer *et al.*, 2014 ▸, 2016 ▸; Danker *et al.*, 2020 ▸). In this case, different SbS_*x*_ species are present in solution, because Schlippesches salt is unstable and forms different reactive species such as [SbS_3_O]^3−^, HS^−^, [S_2_O_3_]^2−^ or [SbS_4_]^3−^ anions (Rammelsberg, 1841 ▸; Long & Bowen, 1970 ▸; Mosselmanns *et al.*, 2000 ▸; Planer-Friedrich & Scheinost, 2011 ▸; Planer-Friedrich & Wilson, 2012 ▸; Anderer *et al.*, 2014 ▸). In addition, a variety of complex redox and condensation reactions occur, generating polymeric thio­anti­monate(III) anions, which are found in the structures of the reaction products. To prevent the reduction of Sb^V^ to Sb^III^, a different synthesis strategy is required and the reaction temperature must be reduced to slow down the decomposition of Schlippesches salt. Using an aqueous solution of Na_3_SbS_4_·9H_2_O and adding a solution of late transition-metal cations leads to immediate precipitation of sulfides or hydroxides, even when chelating amine mol­ecules are added. To solve the problem we developed a two-solution strategy: an organic solution contains the transition-metal cations and the chelating amine mol­ecule and a second solution comprises Schlippesches salt. In the organic solution, the transition-metal complex is already generated *in situ* and mixing the two solutions leads to nucleation and successive growth of the product. A challenge is the integration of transition-metal cations into a thio­anti­monate(V) network, despite the [SbS_4_]^3−^ anion offering four possible binding sites. In the course of this project we became inter­ested in cyclam (cyclam = 1,4,8,11-tetra­aza­cyclo­tetra­deca­ne), which is a tetra­dentate ligand. This means that in an octa­hedral coordination of a transition-metal cation, two coordination sites are provided to which thio­anti­monate(V) anions can coordinate, which, depending on the nature of the anion, can lead to the formation of the desired thio­anti­monate(V) networks. 
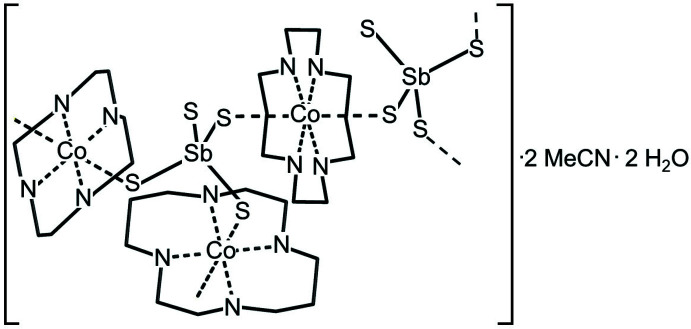


In this context, we have reported on two new polymeric thio­anti­monates with the composition [(Cu-cyclam)_3_(SbS_4_)_2_]·20H_2_O and [(Zn-cyclam)_3_(SbS_4_)_2_]·8H_2_O (Danker *et al.*, 2021 ▸). In the crystal structure of the Cu compound, the copper cations are sixfold coordinated by the four N atoms of the cyclam ligand and two *trans*-sulfur atoms of the [SbS_4_]^3−^ anions within slightly distorted octa­hedra. The copper cations are linked by the anions into rings by corner-sharing SbS_4_ and CuN_4_S_2_ units, which are condensed into layers. These layers are stacked in such a way that large pores are formed. Between the layers, water mol­ecules are embedded. At first glance, the arrangement of the building blocks in the crystal structure of the Zn compound looks similar, but in this case the Zn^II^ cation is disordered above and below the N_4_ plane in a 1:1 ratio, which means that it is in a fivefold coordination of the four N atoms of the cyclam ligand and one S atom of the [SbS_4_]^3−^ anions in a square-pyramidal geometry. The structural difference between the Cu and Zn coordinations was reproduced by DFT calculations (Danker *et al.*, 2021 ▸). In the course of our systematic work we tried to prepare a similar compound with cobalt using the same synthetic approach. This led to crystals of the title compound, which were characterized by single-crystal X-ray diffraction.

## Structural commentary

**Figure 1:**
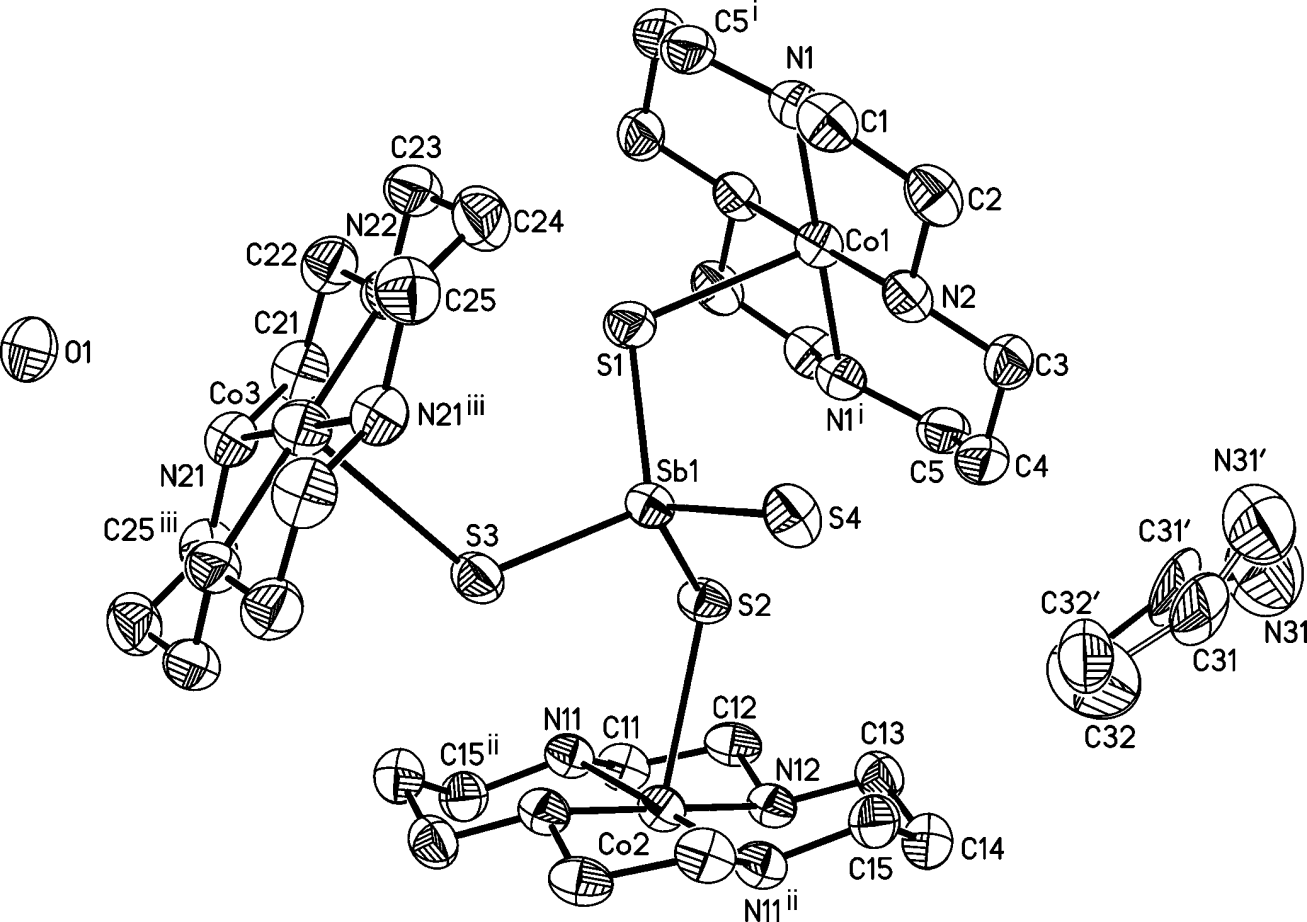
Crystal structure of the title compound with labeling and displacement ellipsoids drawn at the 50% probability level. The hydrogen atoms are omitted for clarity and the disordering of the aceto­nitrile ligands is shown with full and open bonds. Symmetry codes for the generation of equivalent atoms: (i) −*x* + 1, −*y*, −*z* + 2; (ii) −*x* + 1, −*y* + 1, −*z* + 1; (iii) −*x* + 2, −*y*, −*z* + 1.

**Figure 2:**
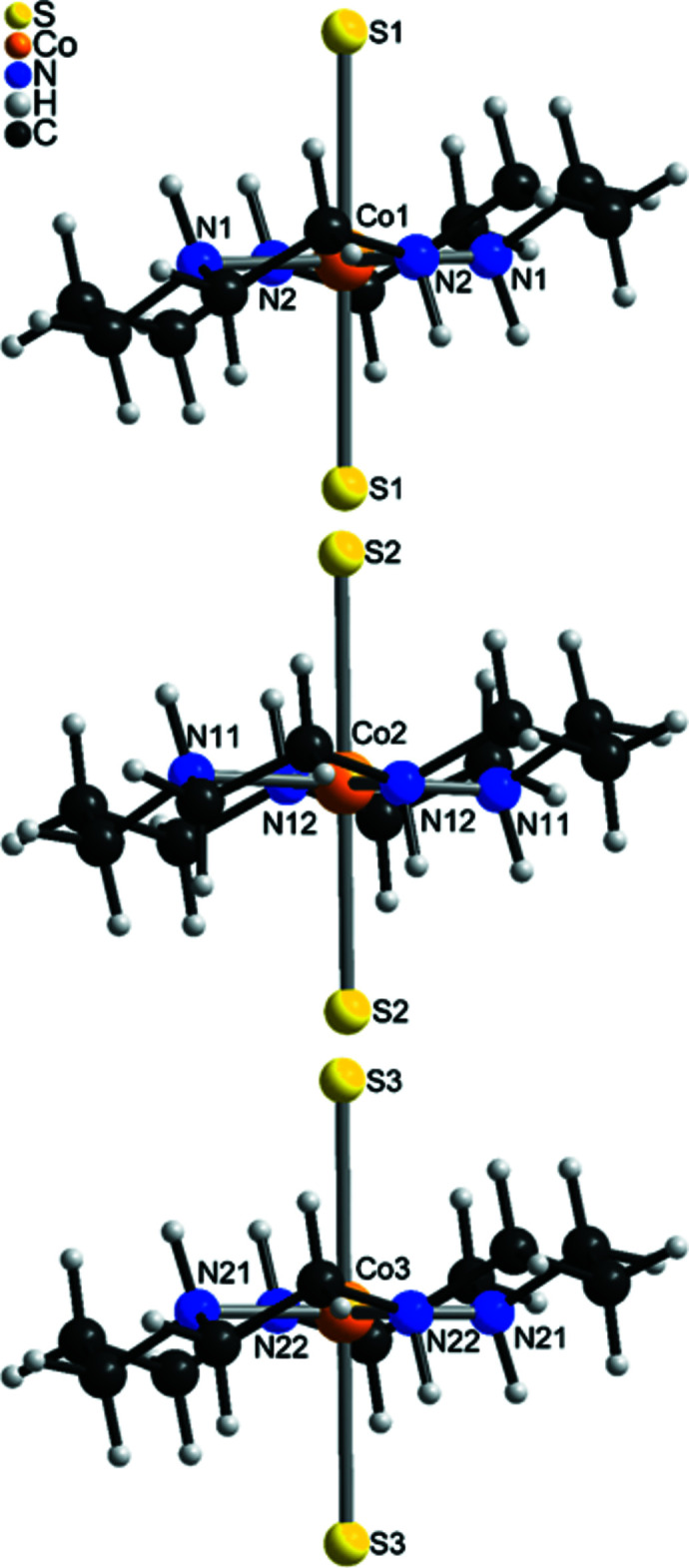
Crystal structure of the title compound with a view of the coordination sphere of the three crystallographically independent Co cations.

The asymmetric unit of the title compound consists of three crystallographically independent Co^II^ cations and three independent cyclam ligands that are located on centers of inversion, as well as one [SbS_4_]^3−^ anion, one water and one aceto­nitrile mol­ecule that occupy general positions (Fig. 1 ▸). The aceto­nitrile mol­ecule is disordered over two orientations and was refined using a split model (see *Refinement*). The Co^II^ cations are six-coordinate being bound to the four N atoms of cyclam ligand that are located in the equatorial plane and two *trans*-S atoms of two inversion-related tetra­thio­anti­monate anions that occupy the apical positions (Fig. 2 ▸). The Co—N bond lengths are very similar for the three crystallographically independent Co^II^ cations whereas significant differences are found for the Co—S bond lengths (Table 1 ▸). These changes, however, do not correlate with the Sb—S distances (Table 1 ▸). The angles around the Co centers prove that the octa­hedra are slightly distorted (see supporting information). The cyclam ligands are in the *trans*-III(*S*,*S*,*R*,*R*) configuration, which is the most stable arrangement for the first row transition-metal cation-centered cyclam complexes (Bosnich *et al.*, 1965 ▸).

**Figure 3:**
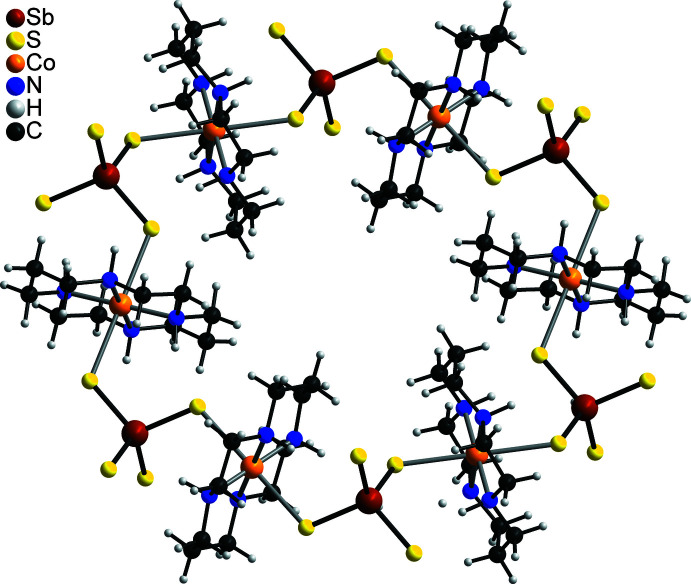
Crystal structure of the title compound with a view of an 24-membered ring composed of six Co cations and six [SbS_4_]^3−^ anions.

**Figure 4:**
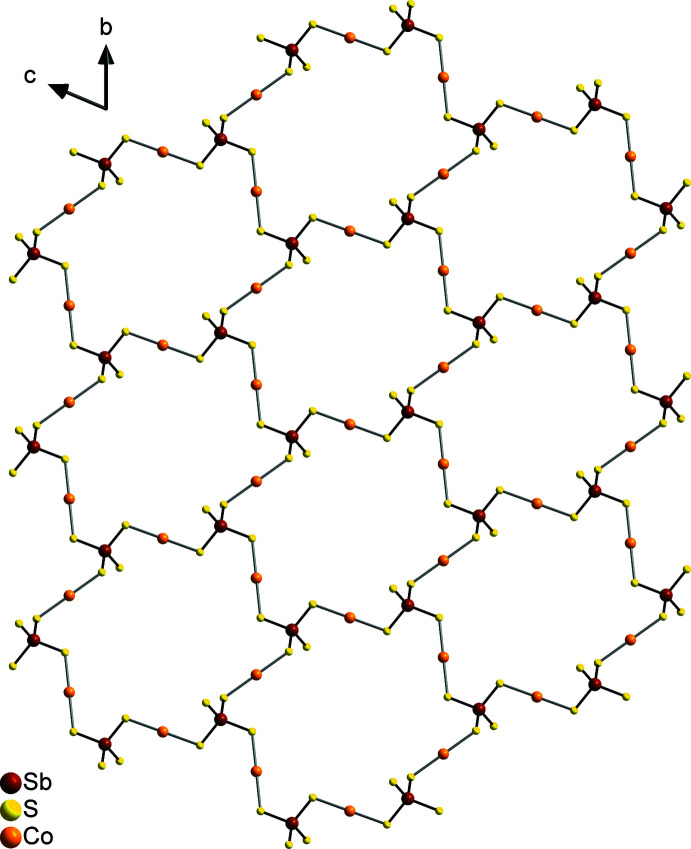
Crystal structure of the title compound with a view of the Co_3_(SbS_4_)_2_ network along the crystallographic *a* axis. The cyclam ligands are not shown for clarity.

**Figure 5:**
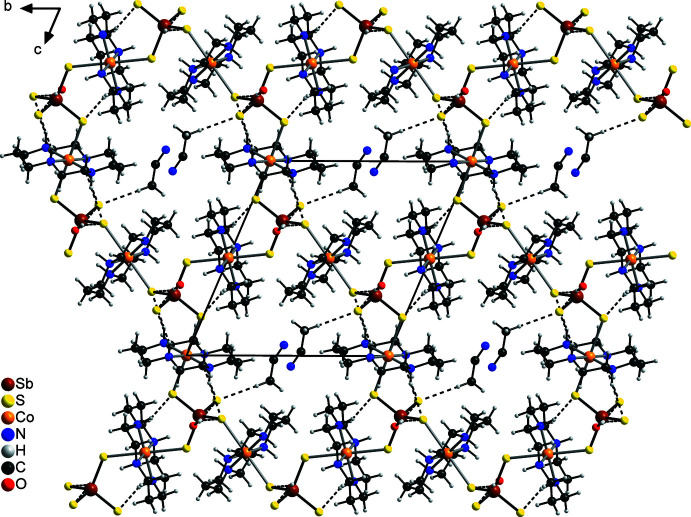
Crystal structure of the title compound with a view in the *a*-axis direction with inter­molecular hydrogen bonding shown as dashed lines. The disorder of the aceto­nitrile mol­ecules is omitted for clarity.

The Sb—S bond lengths in the tetra­thio­anti­monate anion (Table 1 ▸) are comparable and correspond to those observed in other compounds with this anion. From the S—Sb—S bond angles it is obvious that the tetra­hedron is only slightly distorted (see supporting information). The [SbS_4_]^3−^ anion shows the rare tridentate coordination mode and is linked to each of the three crystallographically independent Co^II^ cations and with inversion-related counterparts, forming rings composed of six [SbS_4_]^3−^ anions and six [Co(cyclam]^2+^ cations (Fig. 3 ▸). These rings are condensed into layers parallel to the *bc* plane (Fig. 4 ▸). This layer topology is identical to that in [Cu(cyclam)_3_[SbS_4_)_2_]·20H_2_O but the two compounds are not isotypic (Danker *et al.*, 2021 ▸). The layers are stacked perfectly onto each other, forming channels extending along the a-axis direction (Fig. 5 ▸).

## Supra­molecular features

Within the channels are embedded aceto­nitrile solvate mol­ecules that are disordered and hydrogen bonded to the tetra­thio­anti­monate anion (Fig. 5 ▸). The C—H⋯S angles are close to linear, indicating that this is a significant inter­action (Table 2 ▸). Water mol­ecules are located between the layers and are connected to the [SbS_4_]^3−^ anions *via* inter­molecular O—H⋯S hydrogen bonding, which is classed as strong because the angles are close to linearity and relatively short H⋯S distances are observed (Table 2 ▸). These water mol­ecules also act as acceptors for N—H⋯O hydrogen bonding to the cyclam ligands (Table 2 ▸). The layers are linked by additional C—H⋯S and N—H⋯S hydrogen bonds between the cyclam ligands and the tetra­thio­anti­monate anions. There are additional H⋯S contacts but at distances close to van der Waals contacts with angles ranging between about 110 and 125°.

## Database survey

A search for structures of cobalt-centered cylam complexes in the Cambridge Structural Database (CSD version 5.42, last update November 2020; Groom *et al.*, 2016 ▸) gave 152 hits, in four of which the cobalt cations are in an N_4_S_2_ coordination. In one of these structures (Refcode: NIMVIQ; Zeisler *et al.*, 2013 ▸), a thio­stannate acts as anion but none of them contains thio­anti­monate anions. The same results are obtained if the search is expanded to any transition-metal cation. Therefore, only the Cu and Zn compounds mentioned above have been published (Danker *et al.*, 2021 ▸).

However, 21 structures with Co^II^ and tetra­thio­anti­monate anions have been published and in two of these structures, the cobalt cations are linked to a tetra­thio­anti­monate anion, *viz*. [Co(di­ethyl­enetri­amine)_2_][Co(tris­(2-amino­meth­yl)amine)SbS_4_]_2_·4H_2_O (Engelke *et al.*, 2008 ▸) and [Co(di­ethyl­enetri­am­ine)_2_][Co(tris­(2-amino­meth­yl)amine)SbS_4_]_2_·0.5H_2_O (Lich­te, *et al.*, 2009 ▸).

## Synthesis and crystallization

**Synthesis of Na_3_SbS_4_·9H_2_O**

Na_3_SbS_4_·9H_2_O was synthesized by adding 16.6 g (0.213 mol) of Na_2_S·*x*H_2_O (technical grade, purchased from Acros Organics) to 58 mL of demineralized water. This solution was heated to 323 K for 1 h. Afterwards, 19.6 g (0.058 mol) of Sb_2_S_3_ (98%, purchased from Alfa Aesar) and 3.69 g (0.115 mol) of sulfur (min. 99%, purchased from Alfa Aesar) were added and the reaction mixture was heated to 343 K for 6 h. The reaction mixture was filtered and the filtrate was stored overnight, leading to the formation of slightly yellow crystals, which were filtered off, washed with small amounts of water and dried under vacuum (yield about 30% based on Sb_2_S_3_).

**Synthesis of the title compound**

16 mg (0.044 mmol) of Co(ClO_4_)_2_·6H_2_O (purchased from Alfa Aesar) and 16 mg (0.08 mmol) of cyclam (purchased from Strem Chemicals) were dissolved in 2 mL of aceto­nitrile (purchased from Merck). To this solution, a solution of 50 mg (0.14 mmol) of Na_3_SbS_4_·9H_2_O dissolved in 1 mL of H_2_O was added. Within 3d a few colorless crystals of the title compound were obtained, which were always contaminated with an additional and unknown phase that is amorphous to X-rays. This additional phase is also present if the reaction conditions are varied slightly. Therefore, one of the colorless crystals was selected for structure determination.

## Refinement

Crystal data, data collection and structure refinement details are summarized in Table 3 ▸. All non-hydrogen atoms were refined anisotropically. The C- and N-bound H atoms were located in the difference map but were positioned with ideal­ized geometry (methyl H atoms allowed to rotate but not to tip) and were refined isotropically with *U*_iso_(H) = 1.2*U*_eq_(C,N) (1.5 for methyl H atoms) using a riding model. The O-bound H atoms were located in the difference map, their bond lengths were set to ideal values and finally they were refined isotrop­ically with *U*_iso_(H) = 1.5*U*_eq_(O) using a riding model. The acetontrile mol­ecule is disordered over two orientations and was refined using a split model (ratio: 1:1) with restraints for the geometry and the components of the anisotropic displacement parameters.

## Supplementary Material

Crystal structure: contains datablock(s) I. DOI: 10.1107/S2056989022001074/mw2184sup1.cif

Structure factors: contains datablock(s) I. DOI: 10.1107/S2056989022001074/mw2184Isup2.hkl

CCDC reference: 2146891

Additional supporting information:  crystallographic information; 3D view; checkCIF report

## Figures and Tables

**Table 1:** Selected bond lengths (Å)

Sb1—S4	2.3195 (13)	S1—Co1	2.7258 (12)
Sb1—S1	2.3200 (12)	S2—Co2	2.6932 (11)
Sb1—S3	2.3221 (12)	S3—Co3	2.7821 (12)
Sb1—S2	2.3382 (11)		

**Table 2:** Hydrogen-bond geometry (Å, °)

*D*—H⋯*A*	*D*—H	H⋯*A*	*D*⋯*A*	*D*—H⋯*A*
N1—H1⋯S2^i^	1.00	2.48	3.442 (4)	161
C1—H1*B*⋯S4^ii^	0.99	2.95	3.858 (5)	152
N2—H2⋯S4	1.00	2.49	3.448 (4)	159
N11—H11⋯O1^iii^	1.00	2.23	3.151 (6)	153
N12—H12⋯S3^iv^	1.00	2.43	3.378 (4)	157
N21—H21⋯O1	1.00	2.08	2.920 (6)	141
N22—H22⋯S1	1.00	2.35	3.290 (4)	156
O1—H1*C*⋯S2^iii^	0.84	2.49	3.276 (4)	157
O1—H1*D*⋯S4^v^	0.84	2.46	3.280 (4)	166
C32—H32*B*⋯S4	0.98	2.81	3.71 (4)	154
C32′—H32*F*⋯S4	0.98	2.88	3.85 (5)	172

Symmetry codes: (i) 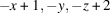 ; (ii) 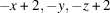 ; (iii) 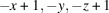 ; (iv) 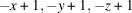 ; (v) 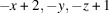 .

**Table 3:** Experimental details

Crystal data	
Chemical formula	[Co_3_(SbS_4_)_2_(C_10_H_24_N_4_)_3_]·2C_2_H_3_N·2H_2_O
*M*_r_	1395.90
Crystal system, space group	Triclinic, *P* 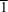
Temperature (K)	200
*a*, *b*, *c* (Å)	8.7292 (3), 12.9680 (5), 13.8936 (5)
α, β, γ (°)	66.218 (3), 77.035 (3), 83.321 (3)
*V* (Å^3^)	1401.93 (9)
*Z*	1
Radiation type	Mo *K*α
μ (mm^−1^)	2.16
Crystal size (mm)	0.15 × 0.10 × 0.07

Data collection	
Diffractometer	Stoe IPDS2
Absorption correction	Numerical (*X-RED* and *X-SHAPE*; Stoe, 2008 ▸)
*T*_min_, *T*_max_	0.649, 0.774
No. of measured, independent and observed [*I* > 2σ(*I*)] reflections	15304, 6098, 5078
*R*_int_	0.030
(sin θ/λ)_max_ (Å^−1^)	0.639

Refinement	
*R*[*F* ^2^ > 2σ(*F* ^2^)], *wR*(*F* ^2^), *S*	0.047, 0.126, 1.06
No. of reflections	6098
No. of parameters	321
No. of restraints	87
H-atom treatment	H-atom parameters constrained
Δρ_max_, Δρ_min_ (e Å^−3^)	0.81, −1.56

Computer programs: *X-AREA* (Stoe, 2008 ▸), *SHELXT* (Sheldrick, 2015*a* ▸), *SHELXL2018/3* (Sheldrick, 2015*b* ▸), *DIAMOND* (Brandenburg, 1999 ▸) and *publCIF* (Westrip, 2010 ▸).

## supplementary crystallographic information

### Crystal data

**Table:**

[Co_3_(SbS_4_)_2_(C_10_H_24_N_4_)_3_]·2C_2_H_3_N·2H_2_O	*Z* = 1
*M**_r_* = 1395.90	*F*(000) = 711
Triclinic, *P*1	*D*_x_ = 1.653 Mg m^−^^3^
*a* = 8.7292 (3) Å	Mo *K*α radiation, λ = 0.71073 Å
*b* = 12.9680 (5) Å	Cell parameters from 15304 reflections
*c* = 13.8936 (5) Å	θ = 1.6–27.0°
α = 66.218 (3)°	µ = 2.16 mm^−^^1^
β = 77.035 (3)°	*T* = 200 K
γ = 83.321 (3)°	Block, red
*V* = 1401.93 (9) Å^3^	0.15 × 0.10 × 0.07 mm

### Data collection

**Table:**

Stoe IPDS-2 diffractometer	5078 reflections with *I* > 2σ(*I*)
ω scans	*R*_int_ = 0.030
Absorption correction: numerical (X-Red and X-Shape; Stoe, 2008)	θ_max_ = 27.0°, θ_min_ = 1.6°
*T*_min_ = 0.649, *T*_max_ = 0.774	*h* = −11→11
15304 measured reflections	*k* = −16→16
6098 independent reflections	*l* = −17→17

### Refinement

**Table:**

Refinement on *F*^2^	Hydrogen site location: mixed
Least-squares matrix: full	H-atom parameters constrained
*R*[*F*^2^ > 2σ(*F*^2^)] = 0.047	*w* = 1/[σ^2^(*F*_o_^2^) + (0.084*P*)^2^ + 0.1597*P*] where *P* = (*F*_o_^2^ + 2*F*_c_^2^)/3
*wR*(*F*^2^) = 0.126	(Δ/σ)_max_ = 0.047
*S* = 1.06	Δρ_max_ = 0.81 e Å^−^^3^
6098 reflections	Δρ_min_ = −1.56 e Å^−^^3^
321 parameters	Extinction correction: *SHELXL2016/6* (Sheldrick 2015b), Fc^*^=kFc[1+0.001xFc^2^λ^3^/sin(2θ)]^-1/4^
87 restraints	Extinction coefficient: 0.0136 (11)

### Special details

**Table:**

Geometry. All esds (except the esd in the dihedral angle between two l.s. planes) are estimated using the full covariance matrix. The cell esds are taken into account individually in the estimation of esds in distances, angles and torsion angles; correlations between esds in cell parameters are only used when they are defined by crystal symmetry. An approximate (isotropic) treatment of cell esds is used for estimating esds involving l.s. planes.

### Fractional atomic coordinates and isotropic or equivalent isotropic displacement parameters (Å^2^)

**Table:**

	*x*	*y*	*z*	*U*_iso_*/*U*_eq_	Occ. (<1)
Sb1	0.73888 (3)	0.19084 (2)	0.69041 (2)	0.03283 (12)	
S1	0.64905 (15)	0.01167 (10)	0.80137 (9)	0.0414 (3)	
S2	0.51359 (13)	0.30840 (9)	0.67415 (9)	0.0380 (3)	
S3	0.86596 (14)	0.19636 (10)	0.52265 (9)	0.0416 (3)	
S4	0.90553 (14)	0.24527 (12)	0.76901 (10)	0.0461 (3)	
Co1	0.500000	0.000000	1.000000	0.0350 (2)	
N1	0.6290 (4)	−0.1388 (3)	1.0629 (3)	0.0392 (8)	
H1	0.583701	−0.171109	1.141506	0.047*	
C1	0.7871 (6)	−0.1025 (5)	1.0528 (4)	0.0461 (11)	
H1A	0.847663	−0.085872	0.979114	0.055*	
H1B	0.843932	−0.162998	1.103578	0.055*	
C2	0.7700 (6)	0.0016 (5)	1.0773 (4)	0.0465 (11)	
H2A	0.720658	−0.016978	1.153611	0.056*	
H2B	0.874555	0.032869	1.063722	0.056*	
N2	0.6701 (5)	0.0849 (3)	1.0071 (3)	0.0391 (8)	
H2	0.736736	0.114228	0.933522	0.047*	
C3	0.6268 (7)	0.1831 (4)	1.0369 (4)	0.0465 (11)	
H3A	0.723689	0.218535	1.033103	0.056*	
H3B	0.567730	0.157340	1.111845	0.056*	
C4	0.5275 (7)	0.2697 (4)	0.9642 (4)	0.0502 (12)	
H4A	0.584251	0.290889	0.889012	0.060*	
H4B	0.515488	0.338203	0.980459	0.060*	
C5	0.3653 (6)	0.2311 (4)	0.9738 (4)	0.0462 (11)	
H5A	0.309991	0.205729	1.049576	0.055*	
H5B	0.304013	0.295721	0.930767	0.055*	
Co2	0.500000	0.500000	0.500000	0.0342 (2)	
N11	0.3450 (4)	0.4238 (3)	0.4693 (3)	0.0375 (8)	
H11	0.350102	0.342674	0.518595	0.045*	
C11	0.1850 (5)	0.4666 (4)	0.5023 (4)	0.0422 (10)	
H11A	0.105455	0.411439	0.515475	0.051*	
H11B	0.160604	0.538662	0.445200	0.051*	
C12	0.1825 (5)	0.4840 (4)	0.6041 (4)	0.0422 (10)	
H12A	0.083455	0.523577	0.622556	0.051*	
H12B	0.188964	0.410337	0.664261	0.051*	
N12	0.3180 (4)	0.5518 (3)	0.5857 (3)	0.0370 (8)	
H12	0.291146	0.630110	0.537533	0.044*	
C13	0.3355 (6)	0.5600 (4)	0.6854 (4)	0.0433 (10)	
H13A	0.348209	0.483093	0.739725	0.052*	
H13B	0.238416	0.594863	0.713526	0.052*	
C14	0.4744 (7)	0.6285 (5)	0.6695 (5)	0.0500 (12)	
H14A	0.471829	0.639111	0.736564	0.060*	
H14B	0.464296	0.703873	0.612383	0.060*	
C15	0.6317 (6)	0.5751 (4)	0.6397 (4)	0.0435 (10)	
H15A	0.717003	0.616669	0.643320	0.052*	
H15B	0.637578	0.496295	0.691914	0.052*	
Co3	1.000000	0.000000	0.500000	0.0441 (2)	
N21	0.8192 (5)	−0.0196 (3)	0.4448 (3)	0.0409 (9)	
H21	0.851395	−0.081969	0.418885	0.049*	
C21	0.6910 (6)	−0.0636 (5)	0.5384 (5)	0.0480 (11)	
H21A	0.610940	−0.097814	0.520863	0.058*	
H21B	0.639594	−0.001829	0.559812	0.058*	
C22	0.7616 (6)	−0.1511 (4)	0.6284 (4)	0.0484 (12)	
H22A	0.680551	−0.178519	0.694862	0.058*	
H22B	0.805027	−0.216022	0.609610	0.058*	
N22	0.8886 (5)	−0.0960 (3)	0.6442 (3)	0.0401 (9)	
H22	0.833782	−0.042725	0.677357	0.048*	
C23	0.9780 (7)	−0.1761 (4)	0.7226 (4)	0.0477 (12)	
H23A	1.027418	−0.235259	0.696736	0.057*	
H23B	0.904885	−0.213090	0.791464	0.057*	
C24	1.1039 (7)	−0.1201 (5)	0.7413 (4)	0.0549 (13)	
H24A	1.148426	−0.175116	0.802670	0.066*	
H24B	1.055165	−0.056814	0.761253	0.066*	
C25	1.2359 (6)	−0.0757 (5)	0.6455 (4)	0.0496 (12)	
H25A	1.320806	−0.049907	0.666612	0.060*	
H25B	1.279912	−0.137189	0.621601	0.060*	
O1	0.7600 (5)	−0.1823 (3)	0.3619 (3)	0.0554 (9)	
H1C	0.704099	−0.205901	0.333522	0.08 (2)*	
H1D	0.850839	−0.202701	0.339072	0.10 (3)*	
N31	0.748 (3)	0.4588 (19)	1.0545 (11)	0.116 (7)	0.5
C31	0.815 (5)	0.457 (4)	0.9743 (13)	0.101 (7)	0.5
C32	0.908 (4)	0.469 (4)	0.8707 (12)	0.090 (7)	0.5
H32A	0.899961	0.547550	0.820009	0.135*	0.5
H32B	0.869880	0.419111	0.844728	0.135*	0.5
H32C	1.018580	0.449588	0.876821	0.135*	0.5
N31'	0.814 (3)	0.4114 (18)	1.0734 (10)	0.112 (7)	0.5
C31'	0.833 (5)	0.452 (4)	0.9817 (11)	0.093 (7)	0.5
C32'	0.854 (5)	0.488 (4)	0.8670 (12)	0.122 (12)	0.5
H32D	0.943593	0.537807	0.831978	0.183*	0.5
H32E	0.758528	0.529186	0.843259	0.183*	0.5
H32F	0.873030	0.422251	0.847836	0.183*	0.5

### Atomic displacement parameters (Å^2^)

**Table:**

	*U*^11^	*U*^22^	*U*^33^	*U*^12^	*U*^13^	*U*^23^
Sb1	0.03142 (17)	0.03401 (17)	0.02939 (17)	−0.00144 (10)	−0.00454 (10)	−0.00920 (11)
S1	0.0463 (6)	0.0347 (5)	0.0360 (6)	−0.0044 (4)	0.0013 (5)	−0.0103 (5)
S2	0.0336 (5)	0.0376 (5)	0.0357 (6)	0.0022 (4)	−0.0059 (4)	−0.0085 (4)
S3	0.0441 (6)	0.0424 (6)	0.0312 (5)	0.0026 (5)	−0.0021 (4)	−0.0111 (5)
S4	0.0384 (6)	0.0608 (7)	0.0406 (6)	−0.0107 (5)	−0.0073 (5)	−0.0189 (6)
Co1	0.0331 (4)	0.0364 (4)	0.0342 (4)	−0.0022 (3)	−0.0069 (3)	−0.0119 (3)
N1	0.0383 (19)	0.041 (2)	0.0338 (19)	0.0015 (16)	−0.0068 (15)	−0.0103 (16)
C1	0.036 (2)	0.055 (3)	0.044 (3)	0.002 (2)	−0.0090 (19)	−0.016 (2)
C2	0.038 (2)	0.061 (3)	0.039 (3)	−0.008 (2)	−0.0099 (19)	−0.016 (2)
N2	0.040 (2)	0.045 (2)	0.0311 (18)	−0.0077 (16)	−0.0064 (15)	−0.0122 (16)
C3	0.060 (3)	0.043 (3)	0.041 (3)	−0.014 (2)	−0.007 (2)	−0.018 (2)
C4	0.065 (3)	0.040 (2)	0.040 (3)	−0.010 (2)	0.000 (2)	−0.013 (2)
C5	0.053 (3)	0.038 (2)	0.038 (2)	0.003 (2)	−0.001 (2)	−0.011 (2)
Co2	0.0295 (4)	0.0378 (4)	0.0331 (4)	−0.0022 (3)	−0.0049 (3)	−0.0118 (3)
N11	0.0363 (19)	0.0381 (19)	0.0348 (19)	−0.0025 (15)	−0.0086 (15)	−0.0095 (16)
C11	0.032 (2)	0.047 (3)	0.044 (3)	−0.0011 (18)	−0.0105 (19)	−0.013 (2)
C12	0.030 (2)	0.042 (2)	0.043 (3)	−0.0043 (17)	−0.0024 (18)	−0.006 (2)
N12	0.0352 (18)	0.0370 (18)	0.0337 (19)	0.0011 (15)	−0.0061 (15)	−0.0093 (15)
C13	0.044 (2)	0.048 (3)	0.035 (2)	0.003 (2)	−0.0039 (19)	−0.016 (2)
C14	0.057 (3)	0.050 (3)	0.046 (3)	−0.004 (2)	−0.008 (2)	−0.022 (2)
C15	0.049 (3)	0.043 (2)	0.043 (3)	−0.003 (2)	−0.015 (2)	−0.017 (2)
Co3	0.0412 (5)	0.0448 (5)	0.0438 (5)	−0.0033 (4)	−0.0067 (4)	−0.0149 (4)
N21	0.0363 (19)	0.042 (2)	0.046 (2)	0.0010 (16)	−0.0086 (16)	−0.0195 (18)
C21	0.035 (2)	0.055 (3)	0.058 (3)	−0.004 (2)	−0.006 (2)	−0.027 (3)
C22	0.046 (3)	0.044 (3)	0.052 (3)	−0.009 (2)	0.003 (2)	−0.020 (2)
N22	0.042 (2)	0.0364 (19)	0.037 (2)	−0.0024 (16)	−0.0023 (16)	−0.0117 (16)
C23	0.056 (3)	0.039 (2)	0.037 (2)	0.003 (2)	−0.002 (2)	−0.009 (2)
C24	0.067 (3)	0.060 (3)	0.042 (3)	0.011 (3)	−0.020 (3)	−0.022 (2)
C25	0.044 (3)	0.058 (3)	0.051 (3)	0.007 (2)	−0.019 (2)	−0.023 (2)
O1	0.054 (2)	0.060 (2)	0.061 (2)	−0.0025 (18)	−0.0136 (19)	−0.031 (2)
N31	0.150 (16)	0.126 (13)	0.073 (4)	−0.026 (10)	0.011 (6)	−0.052 (5)
C31	0.141 (15)	0.097 (11)	0.071 (4)	−0.041 (10)	0.014 (6)	−0.045 (5)
C32	0.117 (15)	0.094 (14)	0.066 (4)	−0.033 (11)	0.002 (6)	−0.041 (7)
N31'	0.162 (19)	0.106 (13)	0.070 (3)	−0.046 (12)	−0.019 (5)	−0.026 (5)
C31'	0.114 (13)	0.100 (13)	0.070 (3)	−0.052 (11)	−0.017 (5)	−0.026 (5)
C32'	0.18 (3)	0.12 (2)	0.070 (3)	−0.07 (2)	−0.020 (6)	−0.026 (5)

### Geometric parameters (Å, º)

**Table:**

Sb1—S4	2.3195 (13)	N12—H12	1.0000
Sb1—S1	2.3200 (12)	C13—C14	1.510 (8)
Sb1—S3	2.3221 (12)	C13—H13A	0.9900
Sb1—S2	2.3382 (11)	C13—H13B	0.9900
S1—Co1	2.7258 (12)	C14—C15	1.514 (7)
S2—Co2	2.6932 (11)	C14—H14A	0.9900
S3—Co3	2.7821 (12)	C14—H14B	0.9900
Co1—N2^i^	1.990 (4)	C15—H15A	0.9900
Co1—N2	1.990 (4)	C15—H15B	0.9900
Co1—N1	1.993 (4)	Co3—N22	1.976 (4)
Co1—N1^i^	1.993 (4)	Co3—N22^iii^	1.976 (4)
N1—C1	1.468 (6)	Co3—N21	1.985 (4)
N1—C5^i^	1.470 (7)	Co3—N21^iii^	1.985 (4)
N1—H1	1.0000	N21—C21	1.472 (7)
C1—C2	1.504 (8)	N21—C25^iii^	1.488 (6)
C1—H1A	0.9900	N21—H21	1.0000
C1—H1B	0.9900	C21—C22	1.506 (8)
C2—N2	1.481 (6)	C21—H21A	0.9900
C2—H2A	0.9900	C21—H21B	0.9900
C2—H2B	0.9900	C22—N22	1.486 (7)
N2—C3	1.476 (6)	C22—H22A	0.9900
N2—H2	1.0000	C22—H22B	0.9900
C3—C4	1.511 (8)	N22—C23	1.468 (6)
C3—H3A	0.9900	N22—H22	1.0000
C3—H3B	0.9900	C23—C24	1.506 (9)
C4—C5	1.517 (8)	C23—H23A	0.9900
C4—H4A	0.9900	C23—H23B	0.9900
C4—H4B	0.9900	C24—C25	1.510 (8)
C5—H5A	0.9900	C24—H24A	0.9900
C5—H5B	0.9900	C24—H24B	0.9900
Co2—N11^ii^	1.975 (4)	C25—H25A	0.9900
Co2—N11	1.975 (4)	C25—H25B	0.9900
Co2—N12	1.985 (4)	O1—H1C	0.8400
Co2—N12^ii^	1.985 (4)	O1—H1D	0.8400
N11—C15^ii^	1.475 (6)	N31—C31	1.145 (15)
N11—C11	1.486 (6)	C31—C32	1.442 (17)
N11—H11	1.0000	C32—H32A	0.9800
C11—C12	1.514 (7)	C32—H32B	0.9800
C11—H11A	0.9900	C32—H32C	0.9800
C11—H11B	0.9900	N31'—C31'	1.145 (15)
C12—N12	1.470 (6)	C31'—C32'	1.442 (16)
C12—H12A	0.9900	C32'—H32D	0.9800
C12—H12B	0.9900	C32'—H32E	0.9800
N12—C13	1.474 (6)	C32'—H32F	0.9800
			
S4—Sb1—S1	109.86 (5)	H12A—C12—H12B	108.4
S4—Sb1—S3	110.64 (5)	C12—N12—C13	111.3 (4)
S1—Sb1—S3	110.81 (5)	C12—N12—Co2	108.0 (3)
S4—Sb1—S2	110.32 (5)	C13—N12—Co2	119.6 (3)
S1—Sb1—S2	105.36 (4)	C12—N12—H12	105.6
S3—Sb1—S2	109.73 (4)	C13—N12—H12	105.6
Sb1—S1—Co1	112.07 (5)	Co2—N12—H12	105.6
Sb1—S2—Co2	122.01 (4)	N12—C13—C14	112.7 (4)
Sb1—S3—Co3	119.94 (5)	N12—C13—H13A	109.0
N2^i^—Co1—N2	180.00 (19)	C14—C13—H13A	109.0
N2^i^—Co1—N1	93.50 (17)	N12—C13—H13B	109.0
N2—Co1—N1	86.50 (17)	C14—C13—H13B	109.0
N2^i^—Co1—N1^i^	86.50 (17)	H13A—C13—H13B	107.8
N2—Co1—N1^i^	93.50 (17)	C13—C14—C15	113.7 (4)
N1—Co1—N1^i^	180.0	C13—C14—H14A	108.8
N2^i^—Co1—S1	89.02 (12)	C15—C14—H14A	108.8
N2—Co1—S1	90.98 (12)	C13—C14—H14B	108.8
N1—Co1—S1	88.50 (12)	C15—C14—H14B	108.8
N1^i^—Co1—S1	91.50 (12)	H14A—C14—H14B	107.7
N2^i^—Co1—S1^i^	90.98 (12)	N11^ii^—C15—C14	111.5 (4)
N2—Co1—S1^i^	89.02 (12)	N11^ii^—C15—H15A	109.3
N1—Co1—S1^i^	91.50 (12)	C14—C15—H15A	109.3
N1^i^—Co1—S1^i^	88.50 (12)	N11^ii^—C15—H15B	109.3
S1—Co1—S1^i^	180.0	C14—C15—H15B	109.3
C1—N1—C5^i^	111.8 (4)	H15A—C15—H15B	108.0
C1—N1—Co1	107.1 (3)	N22—Co3—N22^iii^	180.00 (19)
C5^i^—N1—Co1	119.1 (3)	N22—Co3—N21	86.93 (17)
C1—N1—H1	106.0	N22^iii^—Co3—N21	93.07 (17)
C5^i^—N1—H1	106.0	N22—Co3—N21^iii^	93.07 (17)
Co1—N1—H1	106.0	N22^iii^—Co3—N21^iii^	86.93 (17)
N1—C1—C2	108.2 (4)	N21—Co3—N21^iii^	180.0
N1—C1—H1A	110.1	N22—Co3—S3^iii^	87.74 (11)
C2—C1—H1A	110.1	N22^iii^—Co3—S3^iii^	92.26 (11)
N1—C1—H1B	110.1	N21—Co3—S3^iii^	87.89 (12)
C2—C1—H1B	110.1	N21^iii^—Co3—S3^iii^	92.11 (12)
H1A—C1—H1B	108.4	N22—Co3—S3	92.26 (11)
N2—C2—C1	107.9 (4)	N22^iii^—Co3—S3	87.74 (11)
N2—C2—H2A	110.1	N21—Co3—S3	92.11 (12)
C1—C2—H2A	110.1	N21^iii^—Co3—S3	87.89 (12)
N2—C2—H2B	110.1	S3^iii^—Co3—S3	180.00 (5)
C1—C2—H2B	110.1	C21—N21—C25^iii^	111.9 (4)
H2A—C2—H2B	108.4	C21—N21—Co3	106.4 (3)
C3—N2—C2	111.7 (4)	C25^iii^—N21—Co3	119.8 (3)
C3—N2—Co1	118.9 (3)	C21—N21—H21	105.9
C2—N2—Co1	107.0 (3)	C25^iii^—N21—H21	105.9
C3—N2—H2	106.2	Co3—N21—H21	105.9
C2—N2—H2	106.2	N21—C21—C22	107.7 (4)
Co1—N2—H2	106.2	N21—C21—H21A	110.2
N2—C3—C4	112.0 (4)	C22—C21—H21A	110.2
N2—C3—H3A	109.2	N21—C21—H21B	110.2
C4—C3—H3A	109.2	C22—C21—H21B	110.2
N2—C3—H3B	109.2	H21A—C21—H21B	108.5
C4—C3—H3B	109.2	N22—C22—C21	107.0 (4)
H3A—C3—H3B	107.9	N22—C22—H22A	110.3
C3—C4—C5	114.6 (4)	C21—C22—H22A	110.3
C3—C4—H4A	108.6	N22—C22—H22B	110.3
C5—C4—H4A	108.6	C21—C22—H22B	110.3
C3—C4—H4B	108.6	H22A—C22—H22B	108.6
C5—C4—H4B	108.6	C23—N22—C22	112.3 (4)
H4A—C4—H4B	107.6	C23—N22—Co3	119.9 (3)
N1^i^—C5—C4	112.6 (4)	C22—N22—Co3	106.9 (3)
N1^i^—C5—H5A	109.1	C23—N22—H22	105.6
C4—C5—H5A	109.1	C22—N22—H22	105.6
N1^i^—C5—H5B	109.1	Co3—N22—H22	105.6
C4—C5—H5B	109.1	N22—C23—C24	112.5 (4)
H5A—C5—H5B	107.8	N22—C23—H23A	109.1
N11^ii^—Co2—N11	180.0	C24—C23—H23A	109.1
N11^ii^—Co2—N12	93.73 (16)	N22—C23—H23B	109.1
N11—Co2—N12	86.27 (16)	C24—C23—H23B	109.1
N11^ii^—Co2—N12^ii^	86.27 (16)	H23A—C23—H23B	107.8
N11—Co2—N12^ii^	93.73 (16)	C23—C24—C25	113.8 (5)
N12—Co2—N12^ii^	180.0	C23—C24—H24A	108.8
N11^ii^—Co2—S2^ii^	86.16 (11)	C25—C24—H24A	108.8
N11—Co2—S2^ii^	93.84 (11)	C23—C24—H24B	108.8
N12—Co2—S2^ii^	91.31 (11)	C25—C24—H24B	108.8
N12^ii^—Co2—S2^ii^	88.69 (11)	H24A—C24—H24B	107.7
N11^ii^—Co2—S2	93.84 (11)	N21^iii^—C25—C24	111.6 (4)
N11—Co2—S2	86.16 (11)	N21^iii^—C25—H25A	109.3
N12—Co2—S2	88.69 (11)	C24—C25—H25A	109.3
N12^ii^—Co2—S2	91.31 (11)	N21^iii^—C25—H25B	109.3
S2^ii^—Co2—S2	180.0	C24—C25—H25B	109.3
C15^ii^—N11—C11	111.4 (4)	H25A—C25—H25B	108.0
C15^ii^—N11—Co2	118.1 (3)	H1C—O1—H1D	102.1
C11—N11—Co2	108.6 (3)	N31—C31—C32	172 (5)
C15^ii^—N11—H11	106.0	C31—C32—H32A	109.4
C11—N11—H11	106.0	C31—C32—H32B	109.5
Co2—N11—H11	106.0	H32A—C32—H32B	109.5
N11—C11—C12	107.6 (4)	C31—C32—H32C	109.5
N11—C11—H11A	110.2	H32A—C32—H32C	109.5
C12—C11—H11A	110.2	H32B—C32—H32C	109.5
N11—C11—H11B	110.2	N31'—C31'—C32'	172 (5)
C12—C11—H11B	110.2	C31'—C32'—H32D	109.5
H11A—C11—H11B	108.5	C31'—C32'—H32E	109.5
N12—C12—C11	108.2 (4)	H32D—C32'—H32E	109.5
N12—C12—H12A	110.1	C31'—C32'—H32F	109.4
C11—C12—H12A	110.1	H32D—C32'—H32F	109.5
N12—C12—H12B	110.1	H32E—C32'—H32F	109.5
C11—C12—H12B	110.1		

Symmetry codes: (i) −*x*+1, −*y*, −*z*+2; (ii) −*x*+1, −*y*+1, −*z*+1; (iii) −*x*+2, −*y*, −*z*+1.

### Hydrogen-bond geometry (Å, º)

**Table:**

*D*—H···*A*	*D*—H	H···*A*	*D*···*A*	*D*—H···*A*
N1—H1···S2^i^	1.00	2.48	3.442 (4)	161
C1—H1*B*···S4^iv^	0.99	2.95	3.858 (5)	152
N2—H2···S4	1.00	2.49	3.448 (4)	159
N11—H11···O1^v^	1.00	2.23	3.151 (6)	153
N12—H12···S3^ii^	1.00	2.43	3.378 (4)	157
N21—H21···O1	1.00	2.08	2.920 (6)	141
N22—H22···S1	1.00	2.35	3.290 (4)	156
O1—H1*C*···S2^v^	0.84	2.49	3.276 (4)	157
O1—H1*D*···S4^iii^	0.84	2.46	3.280 (4)	166
C32—H32*B*···S4	0.98	2.81	3.71 (4)	154
C32′—H32*F*···S4	0.98	2.88	3.85 (5)	172

Symmetry codes: (i) −*x*+1, −*y*, −*z*+2; (ii) −*x*+1, −*y*+1, −*z*+1; (iii) −*x*+2, −*y*, −*z*+1; (iv) −*x*+2, −*y*, −*z*+2; (v) −*x*+1, −*y*, −*z*+1.
